# Significant Differences in Thymic Index of Thalassemia Major Patients

**DOI:** 10.4274/tjh.2014.0150

**Published:** 2014-12-05

**Authors:** Yeşim Oymak, Bülent Güzel, Hüseyin Gümüş, Erdem Dağlıoğlu, Ali Ayçiçek, Ahmet Koç, Derya Özyürük

**Affiliations:** 1 Dr. Behçet Uz Children’s Hospital, Clinic of Hematology, İzmir, Turkey; 2 Harran University Faculty of Medicine, Department of Pediatric Hematology, Şanlıurfa, Turkey; 3 Harran University Faculty of Medicine, Department of Radiology, Şanlıurfa, Turkey; 4 Şanlıurfa Children’s Hospital, Clinic of Pediatric Hematology Oncology, Şanlıurfa, Turkey

**Keywords:** Thymus, Blood transfusion, Beta-thalassemia, iron overload, Tymic lndex

## TO THE EDITOR

The thymus can be detected by ultrasonography until the pre-adolescent period. After the early teens, it begins to decrease in size [1]. In thalassemia patients, all organs have the risk of organ dysfunction because of iron overload. Only a few studies showed the size of the thymus in older children and there were no studies in thalassemia patients [2,3]. The side effects of iron overload in thalassemia major (TM) patients’ organs were examined through a literature search, but it appears that the thymus has not been emphasized enough to provide an adequate number of studies. The purpose of this study was to determine whether the size of the thymus in thalassemia patients differed from that in healthy children.

Sixty-five children with TM, aged 1 to 19 years, who had been followed up in the pediatric hematology department at Harran University were enrolled in the study between 1 July and 31 July 2012. Fort-three TM patients and healthy siblings of patients with another diagnosis were enrolled as a control group because there was no literature related to the normal range of thymus size for children older than 8 years.

The thymic size (thymic index) was calculated by multiplying the largest transverse diameter by the largest longitudinal diameter, measured in millimeters by ultrasonography (Toshiba Corporation Medical System Division, Tokyo, Japan, type SSA 240 A with a 7.5-MHz linear probe) [4]. Ultrasonography was performed by the same radiologist. The average ferritin levels for the last 6 months were recorded. 

The study was approved by the Ethics Committee of Harran University. The differences between groups were tested with Mann-Whitney U tests. Correlations were evaluated with Spearman’s test.

**Figure 1:**
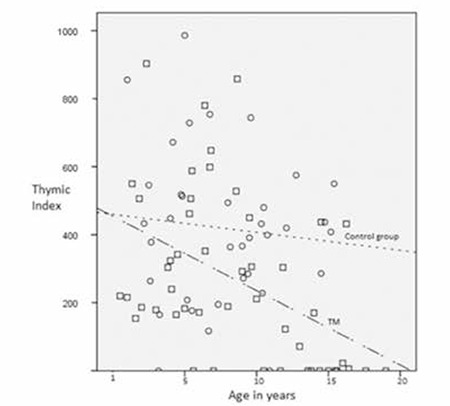
Dot plot of thymic index among children with TM (□—.) and healthy controls (○ ----) at various ages.

The characteristics of the TM patients and the control group are shown in Table 1. There were no differences in terms of sex, age, weight, and height. However, the thymic index of the TM patients was lower than that of the control group (Figure 1).

The TM patients’ mean (± standard deviation) ferritin level was 2967 (±1842) µg/mL. Ferritin level and thymic index did not correlate (r=-0205, p=0.104). Seven (10.7%) TM patients were splenectomized. The median (min-max) thymic index of the splenectomized patients was 0.0 (0.0-304.0); it was 230.5 (0.0-903.0) in the non-splenectomized patients (p=0.001). Splenectomized patients were older than non-splenectomized patients (p=0.001).

In TM patients, the thymic index has not been previously studied. Although corticosteroid effect was eliminated and there were no differences in the age, sex, weight, and height, the thymic index of the TM patients was lower than that of the control group. Iron overload is the best-known harmful effect of chronic transfusion on organs, and it might affect the thymus as well. The size of the thymus is affected by several factors like steroids, infections, and X-rays [5,6,7,8,9]. It has also been found that thymus activity is related to thymus size [10]. In this study, finding a difference in thymus size between TM patients and controls supported the hypothesis that chronic transfusions might affect the thymus; however, there were weaknesses in the groups’ histories in terms of infection, malnutrition, and having undergone X-rays. This is a preliminary study, and the only difference that we knew about was that the groups were receiving chronic blood transfusions, which might contribute to decreased thymus size in TM patients. Zinc level, which is known to be lower in TM patients, may affect the size of the thymus, but there were no data available about the zinc levels of our patients [8]. This study found no association between the thymic index and ferritin level; however, each organ may be affected to a different degree by the same ferritin level [11]. Smaller thymus sizes in splenectomized patients might depend on their older ages.

It was also shown that stress and aging caused thymic involution, which might protect the organism from the danger of autoimmune diseases [12]. In one study it was shown that thymus size declined with age in both children with atopic dermatitis and healthy controls. However, the size of the thymus among children with active atopic dermatitis was higher compared to healthy controls [3]. Having a smaller thymus may be an advantage for TM patients, who are prone to alloimmunization related to transfusion.

In conclusion, thymic involution occurred more rapidly in the TM group than in the normal controls. Further studies that include other parameters, such as T2 magnetic resonance imaging of the thymus for iron load and factors that may affect thymus size, with bigger sample sizes are required to objectively determine the effect of iron overload on thymic involution.

**Conflict of Interest Statement**

The authors of this paper have no conflicts of interest, including specific financial interests, relationships, and/or affiliations relevant to the subject matter or materials included.

## Figures and Tables

**Table 1:**
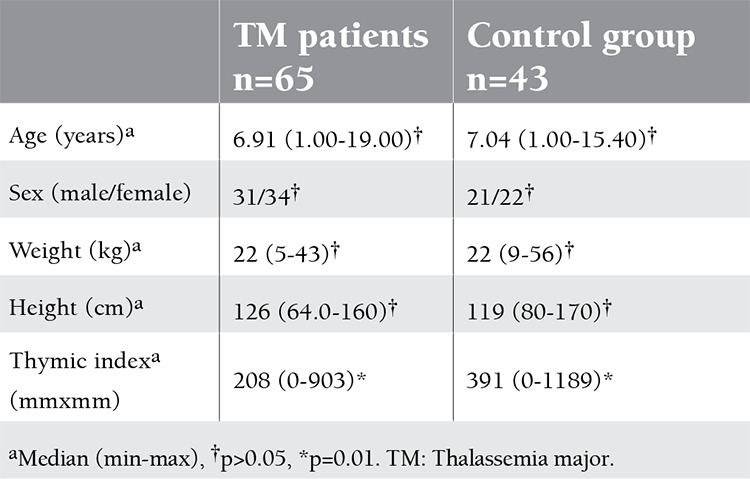
The characteristics of the TM patients and the control group.
